# Telemedicine Practice in Saudi Arabia During the COVID-19 Pandemic

**DOI:** 10.7759/cureus.12004

**Published:** 2020-12-09

**Authors:** Feroze Kaliyadan, Mohammed A. Al Ameer, Ali Al Ameer, Qasem Al Alwan

**Affiliations:** 1 Dermatology, King Faisal University, Al Ahsa, SAU; 2 Dermatology, College of Medicine, King Faisal University, Al Ahsa, SAU; 3 Dermatology, King Fahad General Hospital, Hofuf, SAU; 4 Radiology, King Fahad General Hospital, Hofuf, SAU

**Keywords:** telemedicine, saudi arabia, technology, e-health, covid-19

## Abstract

Objectives: The COVID-19 pandemic has led to an increased use of telemedicine. The primary objective of the study was to evaluate attitudes and behaviors of licensed physicians in the region to telemedicine.

Methodology: A cross-sectional design using an electronic survey as the primary tool was done. The questionnaire had a demographic component of the respondent (first part), covering age, specialty, and experience with telemedicine during the COVID pandemic, and a second part, which was in the form of a Likert scale, covering perceptions related to telemedicine. The Likert scale itself had two main areas: (1) attitudes toward telemedicine and (2) perceived barriers.

Results: There were 392 valid responses of which 228 (58.1%) had used some form of telemedicine (other than standard phone calls) during the COVID-19 pandemic. The most common platforms used for telemedicine include WhatsApp^®^ (211, 53.8%), Zoom^®^ (131, 33.4%), Microsoft Teams^®^ (27, 6.2%), Sehha App (65, 16.5%), Email (84, 21.4%). There was a strong agreement on the following statements: “Telemedicine can reduce unnecessary outpatient visits” (87.5%), “Effectiveness of telemedicine depends on the specialty” (89.5%), and “Telemedicine can be used to monitor chronic patients from home” (88.3%). Concerning the barriers to telemedicine, the ones having the most concordance were technological limitations (66.6%) and concerns of diagnostic reliability (66.1%).

Conclusions: The responses from our study seem to suggest that while the attitudes toward telemedicine are positive, practicing physicians are concerned about a perceived lack of clarity regarding related legal frameworks and barriers such as technological issues, cultural factors, and diagnostic concordance.

## Introduction

The COVID-19 pandemic has led to an increased use of telemedicine all over the world, including Saudi Arabia [1,2]. It is possible that telemedicine will form an integral part of the “new normal.” Although there are some studies that have addressed the practice of telemedicine in Saudi Arabia, these are few and far in between. In general, the actual practice of telemedicine has not been extensive in Saudi Arabia, until recently. The application of telemedicine in Saudi Arabia has seen barriers, especially with respect to acceptance (both from the physician side and the patient side), technology, standardization, and cultural/ethical-legal issues [3-8]. One of the most important barriers that need exploration is the acceptance of telemedicine by practicing physicians. It would therefore be important to study attitudes and perceptions of physicians toward telemedicine. The COVID-19 pandemic has exposed more physicians to actual telemedicine practice. This study evaluates perspectives related to telemedicine from the physician side based on this experience. The results of the same, we feel, will help improve the process and delivery of telemedicine in the long run. The primary objective of the study was to evaluate practices, attitudes, and behaviors of licensed physicians in Saudi Arabia toward telemedicine.

## Materials and methods

The study design was in the form of a cross-sectional survey. An electronic questionnaire was created using Google forms. A team of four physicians (two dermatologists, one radiologist, and one intern) validated the same. The questionnaire had a demographic component covering variables such as age, specialty, grade, and experience with telemedicine during the COVID pandemic. In the second part, perceptions related to telemedicine were evaluated using a Likert scale. The Likert scale itself had two main areas: (1) attitudes toward telemedicine and (2) perceived barriers. The Cronbach’s alpha for the final Likert scale component was an acceptable value of 0.72. The estimated sample size was 384 (Cochran's formula, confidence level 95%, and margin of error 5%). The questionnaire was distributed as a link through social media groups of doctors in the region (response rate was difficult to calculate because of the format of distribution). Descriptive statistics, in the form of frequencies and percentages, are used to represent the data. The study was approved by the Institutional Ethics committee.

## Results

There were 392 valid responses. Age of the respondents ranged from 25 years to 69 years (mean age of 40 years). Of the total, 202 (51.5%) were consultants, 115 (29.3%) were specialists, and the rest being residents. Among the respondents, 305 (77.8%) were conducting routine (live) patient consultations, since the start of the pandemic (the period being about two months); 228 (58.1%) had used some form of telemedicine (other than standard phone calls) during the COVID-19 pandemic; and 188 (47.8%) said that their place of work had dedicated facilities for telemedicine. The most common platforms used for telemedicine were WhatsApp® (211, 53.8%); Zoom® (131, 33.4%); Microsoft Teams® (27, 6.2%); Sehha App, which is a dedicated app developed by the Ministry of Health in Saudi Arabia, (65, 16.5%); and Email (84, 21.4%).

For the component of the questionnaire covering the Likert scale-based responses, there was a strong agreement on the following statements: “Telemedicine can reduce unnecessary outpatient visits” (87.5%), “Effectiveness of telemedicine depends on the specialty”(89.5%), and “Telemedicine can be used to monitor chronic patients from home”(88.3%). A slightly lower level of concordance was seen for the following statements: “Telemedicine is cost-effective” (65.3%), “Telemedicine is an effective tool for providing patient care” (62.5%), and “Patients are satisfied with virtual consultations” (61%). Agreement was relatively lower on the following statements: “The legal aspects of telemedicine practice are clear” (24.2%), and “Telemedicine shows good diagnostic concordance as compared to face-to-face consultations” (27.8%).

As far as the barriers to telemedicine were concerned, the ones having the most concordance were technological limitations (66.6%) and concerns of diagnostic reliability (66.1%). Cultural aspects were also an important aspect according to a large number of respondents (53.3%). Only 36.5% agreed or strongly agreed that physician resistance was a barrier to the practice of telemedicine.

It was interesting that a majority of the respondents agreed or strongly agreed that the use of telemedicine will decrease after the pandemic ends (52.8%).

Details of the Likert scale responses are given in Table 1.

**Table 1:** Responses to the Likert scale component of the questionnaire

n = 392	Strongly disagree	Disagree	Neutral	Agree	Strongly agree
Telemedicine is an effective tool for providing patient care.	15	30	102	160	85
Patients are satisfied with virtual consultations.	8	18	125	194	45
The legal aspects of telemedicine practice are clear.	38	109	150	85	10
Telemedicine is cost-effective.	11	31	94	179	77
There is good scientific evidence for the use of telemedicine.	9	39	179	132	33
Telemedicine shows good diagnostic concordance as compared to face-to-face consultations.	40	123	120	95	14
Telemedicine can reduce unnecessary outpatient visits.	6	12	31	156	187
Effectiveness of telemedicine depends on the specialty.	5	7	29	208	143
Telemedicine can be used to monitor chronic patients from home.	9	8	29	185	161
Which of the following do you think are barriers to the practice of telemedicine? [Physician resistance]	29	106	114	126	17
Which of the following do you think are barriers to the practice of telemedicine? [Patient resistance]	18	62	128	161	23
Which of the following do you think are barriers to the practice of telemedicine? [Diagnostic reliability]	13	19	101	186	73
Which of the following do you think are barriers to the practice of telemedicine? [Cultural aspects]	21	53	109	178	31
Which of the following do you think are barriers to the practice of telemedicine? [Technological limitations]	25	39	67	183	78
The use of telemedicine will decrease after the COVID-19 pandemic is over.	50	68	67	175	32

## Discussion

The COVID-19 pandemic has opened up opportunities to apply, evaluate, and improve the practice of telemedicine [2]. Our study, conducted during the pandemic, gave an opportunity to assess physician attitudes to telemedicine when there was an increased practical application of the same.

The most popular platform for teleconsultations during the pandemic, according to our study, was WhatsApp®. The easy accessibility and ease of sharing data and images are probably what makes this a popular platform. However, there are concerns regarding data confidentiality, misuse, and lack of integration with standard formats like digital imaging and communications in medicine (DICOM) [9].

Previous studies, including systematic reviews, have suggested that telemedicine can be a good triage tool by effectively reducing unnecessary hospital visits and ensuring faster specialist care for patients who need it [10]. The majority of respondents in our study agreed with this concept.

Although patient resistance to telemedicine was an important consideration among our respondents, a majority felt that patient satisfaction to telemedicine is likely to be high. High patient satisfaction with telemedicine has been reported by many studies in the past, including a recent study from Saudi Arabia, conducted during the COVID-19 pandemic [1].

Systematic reviews have also shown how telemedicine can be cost-effective as well as have patient satisfaction scores. The responses in our survey also mirror this opinion. However, studies regarding cost-effectiveness of telemedicine tend to depend on the context, and previous studies have sometimes failed to give conclusive data regarding cost-effectiveness [11-14]. Similarly, studies have shown high patient satisfaction for some contexts but not conclusive data in other contexts [15-17].

Regarding diagnostic concordance and reliability, however, the majority of the respondents in our survey did not agree that telemedicine has high diagnostic concordance. Available literature both contradicts and supports this opinion. However, this could in part be related to the specialty as diagnostic concordance and suitability of telemedicine, in general, are significantly affected by the nature of the specialty [18]. Specialties with inherent visual patient data, like dermatology and radiology, might be better suited to the practice of telemedicine as far as diagnostic concordance is concerned [3].

The lack of clear legal frameworks or the lack of awareness regarding the same is one aspect of telemedicine, which has been highlighted by the respondents in our survey. The same has been echoed in various studies in different areas of the world. Saudi Arabia has recently developed guidelines for the practice of telemedicine [19]. Legal frameworks need to be clear, evaluated, and revised regularly, and the physicians need to be educated on this. The COVID-19 pandemic has led to fast tracking and relaxation of regulations related to telemedicine all over the world [20]. The effects of these changes need to be monitored to ensure protection of patient data and patient safety.

The barriers identified by the respondents in our survey are similar to barriers identified in other studies in different geographical areas. A systematic review of barriers to the application of telemedicine concluded that the main barrier was related to technology. Physicians being resistant to change were another barrier highlighted in this study, as were patient-related factors like age and education. However, the same study suggested that most of these perceived barriers can be overcome by proper, focused training and increased awareness of the involved stakeholders [21]. A positive finding in our study is that physician resistance to change seems to be relatively low. Physician resistance to adopt telemedicine might be due to a combination of factors such as technological issues, lack of clarity on legal frameworks, and concerns regarding patient acceptance and diagnostic reliability. A study from the Eastern Province of Saudi Arabia concluded that the greatest barrier to the implementation of telemedicine was lack of knowledge regarding the same among the stakeholders [6].

A majority of the respondents in our survey felt that local cultural contexts were an important consideration in the implementation of telemedicine. Patients, especially female, might be uncomfortable with video consultations or sharing images for teleconsultations. A previous study has explored the cultural issues with mobile teledermatology. The results of this study indicate that cultural/religious concerns are important in the context of teledermatology. Of 166 patients in the study, 23 patients (14%) refused photography; most of them citing religious reasons [3]. The importance of cultural and legal issues was also highlighted in a systematic review covering telemedicine in Middle Eastern countries [4].

A study from Riyadh also suggested that although the use of smart devices by physicians had increased, there was still some resistance in application of technology for telemedicine [7]. More studies are required to validate telemedicine in the local context. Moreover, although we focused on the practice of telemedicine in our area, attitudes toward telemedicine need to compared and contrasted with similar studies in other countries, with similar sociocultural contexts.

It is possible that COVID-19 will lead to a new normal. The development of telemedicine facilities can be one positive outcome of the pandemic. The COVID-19 pandemic could act as a nidus to standardize, streamline, and optimize the use of telemedicine in our region. The local healthcare system is heavily subsidized, and home monitoring for chronic diseases can significantly reduce financial burden and work force needs on the healthcare system. Focus on specialties more suited for telemedicine (like dermatology and radiology) would be good as an initial step. Evidence-based awareness sessions might overcome many of these perceived barriers to the practice of telemedicine. In the present situation, it might be a good idea to consider making training in telemedicine an integral part of undergraduate medical education, or at least an elective. This will enable early exposure to the theoretical framework of telemedicine and the evidence available for the same.

Government regulation and public-private partnership models to improve technology-related factors and make telemedicine cost-effective will also help in the increasing adoption. In addition, government-level monitoring and evaluation of telemedicine programs are essential to maintain quality.

Limitations

Besides the limitation of being a convenience sampling method, we did not combine the questionnaire with focus-group discussions/structured interviews, which could have improved the quality of the data. We did not incorporate patient feedback into the study design. This would probably have given more meaning to the discussion.

## Conclusions

The responses from our study seem to suggest that there was an increased use of telemedicine during the pandemic. The most popular platform used was WhatsApp®. While the attitudes toward telemedicine were in general positive, practicing physicians are concerned about a perceived lack of clarity regarding related legal frameworks and barriers such as technological issue and diagnostic concordance. Cultural issues also were perceived as a barrier to the practice of telemedicine by most of the respondents. There was a strong agreement that telemedicine could reduce unnecessary outpatient visits and that application of telemedicine depended on the nature of the specialty.

## References

[1] Al-Sofiani ME, Alyusuf EY, Alharthi S, Alguwaihes AM, Al-Khalifah R, Alfadda A (2020). Rapid implementation of a diabetes telemedicine clinic during the coronavirus disease 2019 outbreak: our protocol, experience, and satisfaction reports in Saudi Arabia [IN PRESS]. J Diabetes Sci Technol.

[2] Ashique KT, Kaliyadan F (2020). Teledermatology in the wake of COVID-19 scenario: an Indian perspective. Indian Dermatol Online J.

[3] Kaliyadan F, Amin TT, Kuruvilla J, Ali WHAB (2013). Mobile teledermatology--patient satisfaction, diagnostic and management concordance, and factors affecting patient refusal to participate in Saudi Arabia. J Telemed Telecare.

[4] Al-Samarraie H, Ghazal S, Alzahrani AI, Moody L (2020). Telemedicine in middle eastern countries: progress, barriers, and policy recommendations. Int J Med Inform.

[5] Alaboudi A, Atkins A, Sharp B, Balkhair A, Alzahrani M, Sunbul T (2016). Barriers and challenges in adopting Saudi telemedicine network: the perceptions of decision makers of healthcare facilities in Saudi Arabia. J Infect Public Health.

[6] El-Mahalli AA, El-Khafif SH, Al-Qahtani MF (2012). Successes and challenges in the implementation and application of telemedicine in the eastern province of Saudi Arabia. Perspect Health Inf Manag.

[7] Albarrak AI, Mohammed R, Almarshoud N, Almujalli L, Aljaeed R, Altuwaijiri S, Albohairy T (2019). Assessment of physician's knowledge, perception and willingness of telemedicine in Riyadh region, Saudi Arabia [IN PRESS]. J Infect Public Health.

[8] Alshahrani A, Stewart D, MacLure K (2019). A systematic review of the adoption and acceptance of eHealth in Saudi Arabia: views of multiple stakeholders. Int J Med Inform.

[9] Kaliyadan F, Ashique KT, Jagadeesan S, Krishna B (2016). What's up dermatology? A pilot survey of the use of WhatsApp in dermatology practice and case discussion among members of WhatsApp dermatology groups. Indian J Dermatol Venereol Leprol.

[10] Langabeer JR 2nd, Champagne-Langabeer T, Alqusairi D, Kim J, Jackson A, Persse D, Gonzalez M (2017). Cost-benefit analysis of telehealth in pre-hospital care. J Telemed Telecare.

[11] Chen J, Sun D, Yang W, Liu M, Zhang S, Peng J, Ren C (2018). Clinical and economic outcomes of telemedicine programs in the intensive care unit: a systematic review and meta-analysis. J Intensive Care Med.

[12] Ullah W, Pathan SK, Panchal A (2020). Cost-effectiveness and diagnostic accuracy of telemedicine in macular disease and diabetic retinopathy: a systematic review and meta-analysis. Medicine (Baltimore).

[13] Laver KE, Adey-Wakeling Z, Crotty M, Lannin NA, George S, Sherrington C (2020). Telerehabilitation services for stroke. Cochrane Database Syst Rev.

[14] Snoswell C, Finnane A, Janda M, Soyer HP, Whitty JA (2016). Cost-effectiveness of store-and-forward teledermatology: a systematic review. JAMA Dermatol.

[15] Darcourt JG, Aparicio K, Dorsey PM (2020). Analysis of the implementation of telehealth visits for care of patients with cancer in houston during the COVID-19 pandemic [IN PRESS]. JCO Oncol Pract.

[16] Chaudhry H, Nadeem S, Mundi R (2020). How satisfied are patients and surgeons with telemedicine in orthopaedic care during the COVID-19 pandemic? A systematic review and meta-analysis [IN PRESS]. Clin Orthop Relat Res.

[17] Kozera EK, Yang A, Murrell DF (2016). Patient and practitioner satisfaction with tele-dermatology including Australia's indigenous population: a systematic review of the literature. Int J Womens Dermatol.

[18] Bastola M, Locatis C, Fontelo P (2020). Diagnostic reliability of in-person versus remote dermatology: a meta-analysis [IN PRESS]. Telemed J E Health.

[19] (2020). Telemedicine regulations in the Kingdom of Saudi Arabia. https://nhic.gov.sa/en/Initiatives/Documents/Saudi%20Arabia%20Telemedicine%20Policy.pdf.

[20] Bashshur R, Doarn CR, Frenk JM, Kvedar JC, Woolliscroft JO (2020). Telemedicine and the COVID-19 pandemic, lessons for the future. Telemed J E Health.

[21] Scott Kruse C, Karem P, Shifflett K, Vegi L, Ravi K, Brooks M (2018). Evaluating barriers to adopting telemedicine worldwide: a systematic review. J Telemed Telecare.

